# Estimation of Unreported Novel Coronavirus (SARS-CoV-2) Infections from Reported Deaths: A Susceptible–Exposed–Infectious–Recovered–Dead Model

**DOI:** 10.3390/jcm9051350

**Published:** 2020-05-05

**Authors:** Andrea Maugeri, Martina Barchitta, Sebastiano Battiato, Antonella Agodi

**Affiliations:** 1 Department of Medical and Surgical Sciences and Advanced Technologies “GF Ingrassia”, University of Catania, 95123 Catania, Italy; andrea.maugeri@unict.it (A.M.); martina.barchitta@unict.it (M.B.); 2 Department of Mathematics and Computer Science, University of Catania, 95123 Catania, Italy; battiato@dmi.unict.it; 3 Azienda Ospedaliero-Universitaria “Policlinico-Vittorio Emanuele”, 95123 Catania, Italy

**Keywords:** novel coronavirus, COVID-19, epidemic model, epidemiology

## Abstract

In the midst of the novel coronavirus (SARS-CoV-2) epidemic, examining reported case data could lead to biased speculations and conclusions. Indeed, estimation of unreported infections is crucial for a better understanding of the current emergency in China and in other countries. In this study, we aimed to estimate the unreported number of infections in China prior to the 23 January 2020 restrictions. To do this, we developed a Susceptible–Exposed–Infectious–Recovered–Dead (SEIRD) model that estimated unreported infections from the reported number of deaths. Our approach relied on the fact that observed deaths were less likely to be affected by ascertainment biases than reported infections. Interestingly, we estimated that the basic reproductive number (*R_0_*) was 2.43 (95%CI = 2.42–2.44) at the beginning of the epidemic and that 92.9% (95%CI = 92.5%–93.1%) of total cases were not reported. Similarly, the proportion of unreported new infections by day ranged from 52.1% to 100%, with a total of 91.8% (95%CI = 91.6%–92.1%) of infections going unreported. Agreement between our estimates and those from previous studies proves that our approach is reliable for estimating the prevalence and incidence of undocumented SARS-CoV-2 infections. Once it has been tested on Chinese data, our model could be applied to other countries with different surveillance and testing policies.

## 1. Introduction

The novel coronavirus (SARS-CoV-2) outbreak, which spread in Wuhan (Hubei Province, China) at the end of 2019, has caused 81,554 cases and 3312 deaths among the Chinese population as of 1 April 2020 [1]. Whilst the number of SARS-CoV-2 infections is decreasing in China, other countries are still facing the epidemic and global efforts to contain the virus are still ongoing [1]. However, given the uncertainty about the transmissibility and virulence of SARS-CoV-2, the effectiveness of strategies against the current epidemic should be assessed properly [2]. In this scenario, the proportion of unreported infections is particularly noteworthy due to its crucial role in modulating the spread of the virus [2]. Indeed, unrecognized cases—often patients who experience mild or no symptoms—could silently expose a far greater proportion of the population to SARS-CoV-2 [3]. Correspondingly, it has recently been estimated that the transmission rate of undocumented infections was about half of those documented, and that undocumented infections could be the source of eight out of ten documented cases [2]. Several countries are implementing stringent testing strategies for severely ill patients or those who have come into contact with documented cases [4]. This could lead to losing track of mild or asymptomatic patients who, however, could be infectious [5]. Therefore, looking only at reported case data could lead to biased speculations and hasty conclusions. In contrast, observed deaths are less likely to be affected by ascertainment biases, with the exception of deaths in the early phase of the epidemic [5].

For these reasons, we hypothesized that we could estimate the unreported number of infections by working directly with reported deaths. We employed a Susceptible–Exposed–Infectious–Recovered–Dead (SEIRD) model to estimate the number of unreported infections of SARS-CoV-2 in China prior to 23 January 2020, the date on which China imposed a lockdown in Wuhan and other cities of Hubei province in an effort to quarantine the epicenter of the SARS-CoV-2 outbreak.

## 2. Materials and Methods

**Figure 1:**
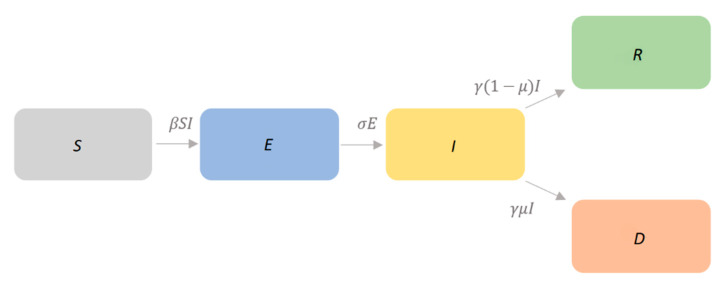
The employed Susceptible–Exposed–Infectious–Recovered–Dead (SEIRD) epidemic model for SARS-CoV-2. *β*, *σ*; *γ*, and *μ* denote the transmission rate, infection rate, removing rate, and probability of infectious individuals dying, respectively. *S*, *E*, *I*, *R*, and *D* denote susceptible, exposed, infectious, recovered, and dead individuals, respectively.

We used available public data on the daily number of cases and deaths in China released by the European Centre for Disease Prevention and Control [6]. All cases were laboratory confirmed following the case definition by the National Health Commission of China [6]. In line with previous studies [7,8,9,10,11], a Susceptible-Exposed-Infectious-Removed (SEIR) model was exploited but care was also taken to separate the removed state into two classes: recovered cases (R) and deaths (D). Indeed, in the traditional SEIR model, the removed state ideally includes both recovered and dead patients. In our study, however, we aimed to estimate the number of deaths through the SEIRD model and to fit the model itself to the reported number of deaths. A visual summary of our model is displayed in Figure 1.

In particular, the model was defined by the following ordinary differential equations:

$$\frac{dS\left(t\right)}{dt}=-\frac{\beta S\left(t\right)I\left(t\right)}{N}$$

$$\frac{dE\left(t\right)}{dt}=\frac{\beta S\left(t\right)I\left(t\right)}{N}-\sigma E\left(t\right)$$

$$\frac{dI\left(t\right)}{dt}=\sigma E\left(t\right)-\gamma I\left(t\right)$$

$$\frac{dR\left(t\right)}{dt}=\gamma \left(1-\mu \right)I\left(t\right)$$

$$\frac{dD\left(t\right)}{dt}=\gamma \mu I\left(t\right)$$

where:

- *S(t), E(t), I(t)*, *R(t)*, and *D(t)* are the numbers of susceptible, exposed (infected but not yet infectious), infectious, recovered, and dead individuals at the time *(t)*;
- *N* is the total population as *N = S + E + I + R + D*. Note that the model relies on $\frac{S}{N}$ and hence is not affected by increasing *N*;
- *β* is the transmission rate, also known as the effective contact rate;
- *σ* is the infection rate and was assumed to be the inverse of the incubation period (i.e., the period from infection to the onset of symptoms);
- *γ* is the removing rate and was assumed to be the inverse of the period between the onset of symptoms and recovery/death;
- *μ* is the probability of infectious individuals dying.

**Figure 2:**
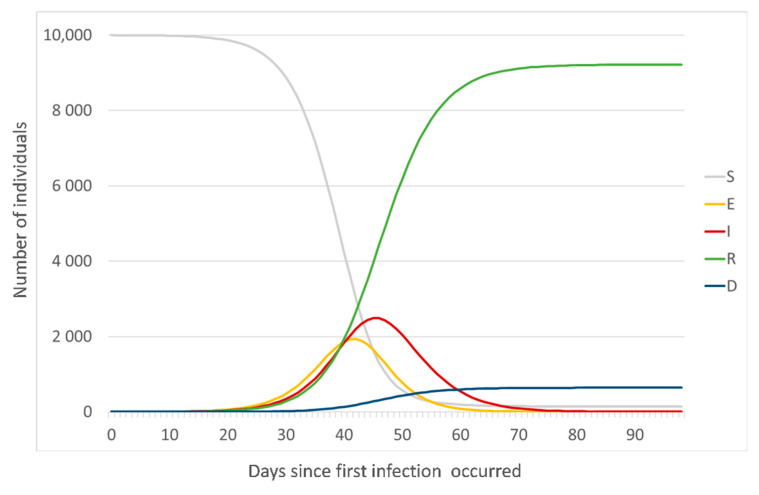
Generic representation of SEIRD states along the temporal axis. Estimates were obtained through a SEIRD model with *β, σ, γ*, and *μ* set as 0.8, 0.3, 0.2, and 0.2, respectively (for viewing purposes only).

Figure 2 depicts, as an example, the number of individuals in each state since an infection occurred in a population of 10,000 individuals. The graph was obtained through a generic SEIRD model with *β, σ, γ*, and *μ* set as 0.8, 0.3, 0.2, and 0.2, respectively.

In the current study, *N* was assumed to be 1 billion, *R* and *D* were initially set as 0, while the initial number of infectious individuals was set to 1. In the early phase of the epidemic, it was not possible to completely exclude a small fraction of undocumented deaths. Moreover, given the lag of 2–3 weeks between transmission changes and their impact on mortality trends, we were very confident in using data within 2 weeks after the travel restrictions. For these reasons, we fitted our model to the reported number of deaths from 23 January (i.e., the day after China had cumulatively observed 10 deaths) to 7 February. In the baseline scenario, we assumed *σ* and *γ* as 1/5.2 days and 1/3.5 days, respectively, according to previous studies [2,3]. The initial ranges of the unknown model parameters were 0.1 ≤ *β* ≤ 1 and 0.001 ≤ *μ* ≤ 0.200, respectively.

To estimate unknown parameters with their 95% confidence interval (95%CI), which best explained the reported number of deaths, we applied a least squares optimization using an evolutionary algorithm (population size = 1 × 10^5^, convergence = 1 × 10^−6^, and mutation rate = 5 × 10^−2^) and simulations (n = 1000) on randomly generated samples from the cumulative distribution function of reported deaths. Estimated infections and total cases from 31 December to 23 January were obtained from the best-fitting SEIRD model. The values of unreported new infections and total cases were obtained by subtracting the reported numbers from those estimated and are reported as a percentage. The basic reproductive number (*R_0_*) was calculated from the SEIRD model as previously described [12]. We also performed sensitivity analyses to evaluate the impact of varying the infectious period and the initial number of infectious individuals on the estimation of unreported cases and infections.

## 3. Results

**Figure 3:**
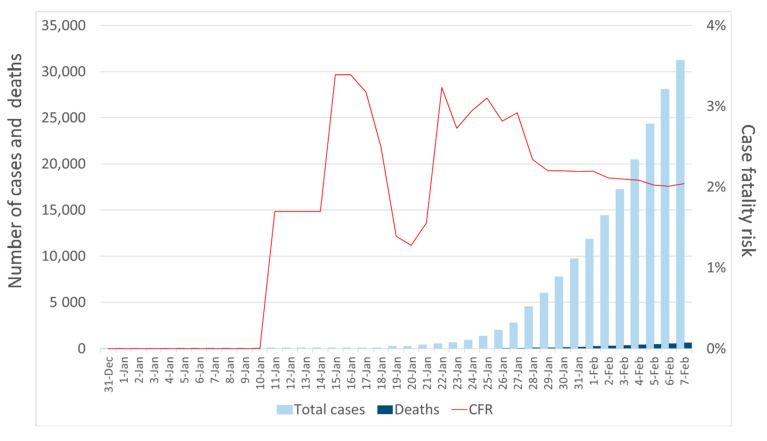
Number of reported cases and deaths in China from 31 December 2019 to 7 February 2020. The bars represent the cumulative number of reported coronavirus (SARS-CoV-2) cases and related deaths while the red line represents the case fatality risk (CFR).

The cumulative number of cases and deaths by the day of the report, from 31 December 2019 to 7 February 2020, are shown in Figure 3. Looking at the case fatality risk (i.e., the number of deaths in persons who tested positive for SARS-CoV-2 divided by number of SARS-CoV-2 cases), we noted high fluctuations that could be attributed to the proportion of unreported cases or deaths. However, as previously discussed, observed deaths were less prone to be affected by ascertainment biases than documented cases.

**Figure 4:**
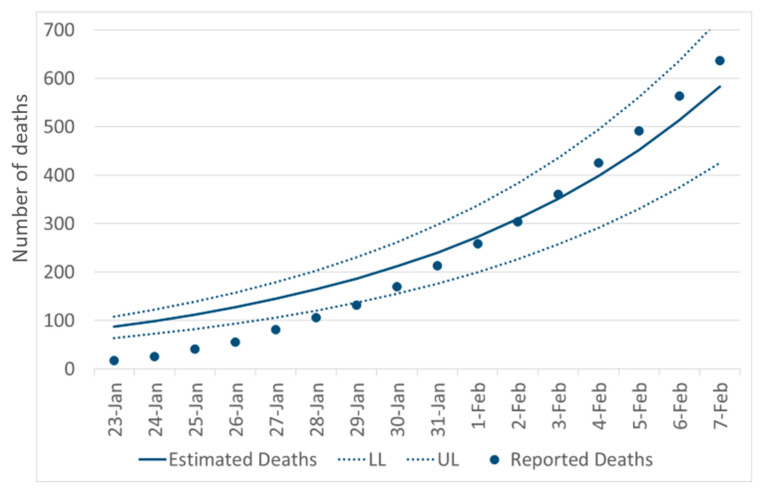
Fitting the SEIRD model to the reported number of deaths. The dots represent the daily cumulative number of reported deaths while the lines along the temporal axis represent the estimate and 95% confidence intervals (Upper and Low Level: UL, LL) through the SEIRD model.

Accordingly, we first fitted our SEIRD model to reported deaths (Figure 4), which suggested an overall good fit between estimated and reported deaths (Correlation Coefficient *R^2^* = 0.987). The slight over-prediction in the early phase of our modeling was likely due to a still existing proportion of undocumented deaths among SARS-CoV-2 cases.

**Figure 5:**
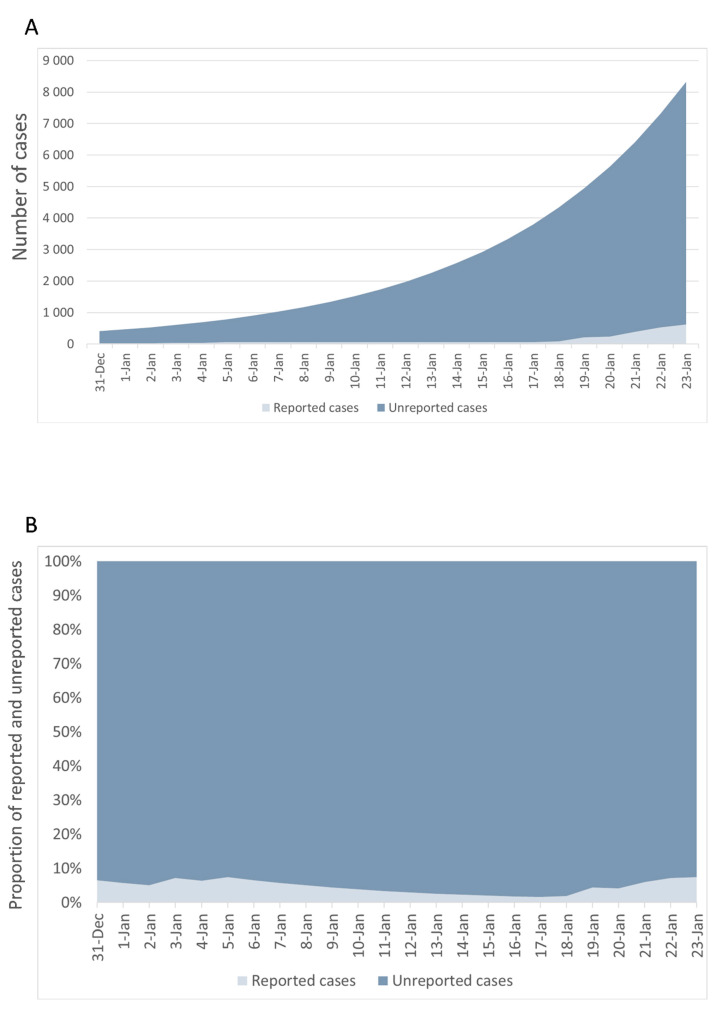
Estimated number of cases (**A**) and proportion of unreported events (**B**) from 31 December 2019 to 23 January 2020.

Using the best-fitting parameters reported in Table 1, we estimated that the *R_0_* was 2.43 (95%CI = 2.42–2.44) with a total of 8724 (95%CI = 8478–8921) estimated cases on 23 January 2020. These estimates and their comparison with reported cases (Figure 5) revealed 8101 (95%CI = 7855–8298) unreported cases, which represented 92.9% (95%CI = 92.5%–93.1%) of estimated cases.

**Figure 6:**
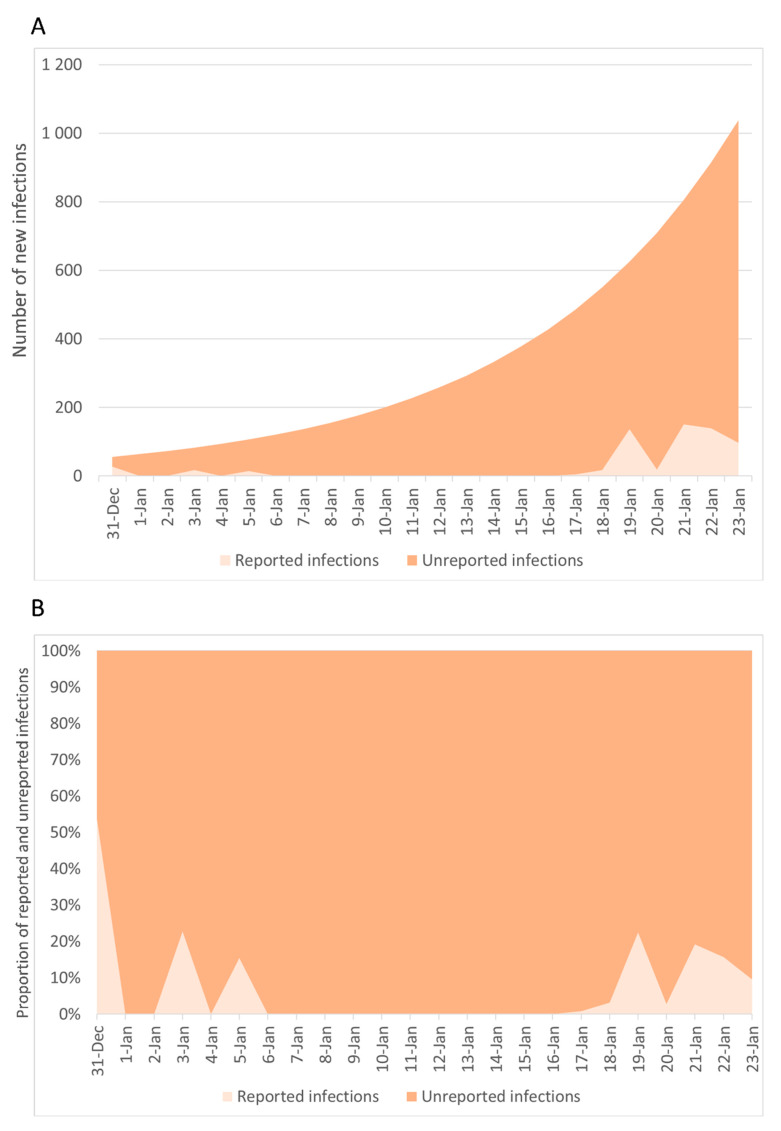
Estimated number of new infections (**A**) and proportion of unreported events (**B**) from 31 December 2019 to 23 January 2020.

Accordingly, the estimated number of new infections from 31 December 2019 to 23 January 2020 was 8307 (95%CI = 8069–8498) (Figure 6). The proportion of unreported new infections by day ranged from 52.1% to 100%, which resulted in a total of 7684 (95%CI = 7446–7875) unreported new infections and a proportion of 91.8% (95%CI = 91.6%–92.1%).

Given that the removing rate was one of the most debated epidemic parameters—with previous estimates ranging from 3 to 20 days—we performed a sensitivity analysis where we fitted the SEIRD model with different *γ* values. However, neither estimated values nor unreported proportions were sensitive to changes in the removing rate (Supplementary Figures S1–S4). Instead, the *R_0_* would increase to 4.07 (95%CI = 3.91–4.17) or 6.50 (95%CI = 6.45–6.55) if we assumed *γ* to be 0.1 and 0.05, respectively. Similarly, we analyzed the condition where the initial number of infectious individuals was 100 times greater than the baseline scenario. Nevertheless, the estimates were not sensitive to changes, while the *R_0_* decreased to 1.60 (95%CI = 1.45–1.76) (Supplementary Figures S5–S6**)**.

## 4. Discussion

In this study, we estimated the unreported number of SARS-CoV-2 cases in China prior to the 23 January 2020 lockdown. Our estimates reveal a very high proportion of unreported new infections every day, which resulted in 92.9% unreported cases. This finding was almost aligned with other recent estimates of unreported infections for the same time period [2,13]. For instance, Li and colleagues [2] reported that 86% of all infections were undocumented prior to travel restrictions, and that the transmission rate of undocumented infections was approximately 50% of documented infections. Yet, we obtained similar estimates by using a modified SEIR model, which took into account dead individuals in the removed state. To the best of our knowledge, our study was the first that applied a SEIRD model to estimate the number of infections from observed deaths. Only a few research groups are investigating the SARS-CoV-2 epidemic curve by calculating backwards from the deaths observed over time [5]. Our findings were also corroborated by the estimated *R_0_*, approximately 2.4, which was consistent with previous estimates [2,5,9,14,15] and which indicated a high capacity for sustained transmission at the beginning of the epidemic.

Our study has some limitations. First, our hypothesis was that data on deaths were less likely to be affected by under-reporting than data on infections, given that the proportion of deaths among mild or asymptomatic patients was supposed to be lower [4]. The number of deaths, however, was not exempt from ascertainment issues. Indeed, clear criteria for the definition of SARS-CoV-2-related deaths were not available [4], and thus it might be possible that some deaths were caused by pre-existing conditions rather than this infection. Nevertheless, our model did not rely on a causal relationship between SARS-CoV-2 infection and deaths but only on the probability of dying among infectious individuals (i.e., *μ*). This parameter, along with the removing rate (i.e., *γ*), regulated the transition of infectious individuals to death. We also recognized that our approach relied on several assumptions and that many parameters had to be fixed. However, we have provided reasonable grounds and relevant citations to previous studies and performed a sensitivity analysis for those parameters that required further investigations. Nevertheless, sensitivity analyses made using alternative γ values or increasing the initial number of infectious individuals gave similar estimates of unreported cases but different values of the *R_0_*. Given this, we cannot rule out some degree of uncertainty from our estimates; however, they will be more reliable as more data become available.

In conclusion, our estimates are important for a better understanding of the SARS-CoV-2 epidemic in China and in other countries. Our approach, based on the observed deaths, has proven to be reliable for estimating the prevalence and incidence of undocumented SARS-CoV2 infections. Thus, our model could be applied in other countries with different surveillance and testing policies, and partially explains, for instance, differences in epidemic transmission and case fatality risk worldwide.

## Figures and Tables

**Table 1:** Initial conditions, assumptions, and best-fitting parameters in the baseline scenario.

SEIRD Parameters	Definition	Assumed or Estimated Parameters
***β*^a^**	Transmission rate	0.73 (95%CI = 0.72–0.74)
***σ*^b^**	Infection rate	0.19
***γ*^c^**	Removing rate	0.28
***μ*^d^**	Probability of dying	0.015 (95%CI = 0.011–0.018)

^a^ Estimated through the model with a potential range of 0.1 ≤ *β* ≤ 1.0. ^b^ Assumed to be $\frac{1\;}{5.2}\;$days according to Li and colleagues [3]. ^c^ Assumed to be $\frac{1}{3.5}\;$days according to Li and colleagues [2]. ^d^ Estimated through the model with a potential range of 0.01 ≤ *μ* ≤ 0.20.

## Supplementary Materials

The following are available online at https://www.mdpi.com/2077-0383/9/5/1350/s1, Figure S1: Estimated number of cases (A) and proportion of unreported events (B) from 31 December 2019 to 23 January 2020 using γ = 0.1; Figure S2: Estimated number of cases (A) and proportion of unreported events (B) from 31 December 2019 to 23 January 2020 using γ = 0.05; Figure S3: Estimated number of new infections (A) and proportion of unreported events (B) from 31 December 2019 to 23 January 2020 using γ = 0.1; Figure S4: Estimated number of new infections (A) and proportion of unreported events (B) from 31 December 2019 to 23 January 2020 using γ = 0.05; Figure S5: Estimated number of cases (A) and proportion of unreported events (B) from 31 December 2019 to 23 January 2020 using an initial infectious individuals number of 100; Figure S6: Estimated number of new infections (A) and proportion of unreported events (B) from 31 December 2019 to 23 January 2020 using an initial infectious individuals number of 100.
