# Supplementary material for: Estimation of Unreported Novel Coronavirus (SARS-CoV-2) Infections from Reported Deaths: A Susceptible–Exposed–Infectious–Recovered–Dead Model
Source: J Clin Med. 2020 May 5;9(5):1350. doi: 10.3390/jcm9051350 (PMC7291317; doi:10.3390/jcm9051350)
Supplement: Supplementary file 1 [file jcm-09-01350-s001.zip › jcm-779604-supplementary.docx]

**Supplementary Materials**

**Estimation of Unreported Novel Coronavirus (SARS-CoV-2) Infections from Reported Deaths: a Susceptible-Exposed-Infectious-Recovered-Dead model**

Andrea Maugeri, Martina Barchitta, Sebastiano Battiato and Antonella Agodi

**Figure S1.** Estimated number of cases (A) and proportion of unreported events (B) from 31 December 2019 to 23 January 2020 using γ = 0.1

**Figure S2.** Estimated number of cases (A) and proportion of unreported events (B) from 31 December 2019 to 23 January 2020 using γ = 0.05

**Figure S3.** Estimated number of new infections (**A**) and proportion of unreported events (**B**) from 31 December 2019 to 23 January 2020 using γ = 0.1

**Figure S4.** Estimated number of new infections (**A**) and proportion of unreported events (**B**) from 31 December 2019 to 23 January 2020 using γ = 0.05

**Figure S5.** Estimated number of cases (**A**) and proportion of unreported events (**B**) from 31 December 2019 to 23 January 2020 using an initial infectious individuals number of 100

**Figure S6.** Estimated number of new infections (**A**) and proportion of unreported events (**B**) from 31 December 2019 to 23 January 2020 using an initial infectious individuals number of 100

**Figure S1.** Estimated number of cases (**A**) and proportion of unreported events (**B**) from 31 December 2019 to 23 January 2020 using γ = 0**Figure S2.** Estimated number of cases (**A**) and proportion of unreported events (**B**) from 31 December 2019 to 23 January 2020 using γ = 0.05

** Figure S3.** Estimated number of new infections (**A**) and proportion of unreported events (**B**) from 31 December 2019 to 23 January 2020 using γ = 0.1

**Figure S4.** Estimated number of new infections (**A**) and proportion of unreported events (**B**) from 31 December 2019 to 23 January 2020 using γ = 0.05

**Figure S5.** Estimated number of cases (**A**) and proportion of unreported events (**B**) from 31 December 2019 to 23 January 2020 using an initial infectious individuals number of 100

**Figure S6.** Estimated number of new infections (**A**) and proportion of unreported events (**B**) from 31 December 2019 to 23 January 2020 using an initial infectious individuals number of 100
